# Current Indications and Techniques for the Use of Bowel Segments in Pediatric Urinary Tract Reconstruction

**DOI:** 10.3389/fped.2019.00236

**Published:** 2019-06-12

**Authors:** Raimund Stein, Katrin Zahn, Nina Huck

**Affiliations:** Department of Pediatric, Adolescent and Reconstructive Urology, Medical Faculty Mannheim, Medical Center Mannheim, Heidelberg University, Mannheim, Germany

**Keywords:** urinary diversion, urinary diversion complication, urinary diversion-methods, children and adolescent, surgical complications

## Abstract

Today, there are few indications for the use of bowel in pediatric urology. This is in large extent due to the successful conservative therapy in patients with neurogenic bladder and the improved success of primary reconstruction in patients with the bladder exstrophy-epispadias complex. Only after the failure of the maximum of conservative therapy or after failure of primary reconstruction, bladder augmentation, or urinary diversion should be considered. Malignant tumors of the lower urinary tract (e.g., rhabdomyosarcomas of the bladder/prostate) are other rare indications for urinary diversion. Replacement or reconstruction of the ureter with a bowel segment is also a quite rarely performed procedure. In this review, the advantages and disadvantages of the different options for the use of bowel segments for bladder augmentation, bladder substitution, urinary diversion, or ureter replacement during childhood and adolescence are discussed.

## Introduction

Today, the indication for the use of bowel segments in pediatric and adolescent urology for bladder augmentation, substitution, or continental urinary diversion has been markedly decreased.

Nowadays, the establishment of early conservative therapy with intermittent catheterization and pharmacotherapy in patients with a neurogenic bladder due to spina bifida seems to lead to a reduction in surgical therapy—at least early in life (1–3). After establishment of the conservative treatment, the numbers of augmentation did not decrease any more, at least in the USA (4, 5). In patients with bladder exstrophy or incontinent epispadias, primary reconstruction has become the most accepted approach (6, 7). If conservative therapy or primary reconstruction does not lead to the desired outcome or if the function of the upper urinary tract is endangered, bladder augmentation, or urinary diversion should be considered (8). The indication for radical cystectomy is extremely rare in children and adolescents (9). The replacement of the ureter in this age group is even more rare (10–18).

Considering the use of bowel segments, the special situation of the patients and their family's abilities and conditions, previous operative interventions and last but not least the expectations and wishes of the patient, their families and the surgeon must be considered. It is also essential, that the patient and parents have to be informed in detail about the advantages and disadvantages of the various forms of urinary diversion, their surgical complications and metabolic consequences (2, 19). Furthermore, it is advantageous, that an uro-therapist or stoma-therapist supervises the patients right from the beginning and trains the postoperative care in detail. This is the best way to respond to unrealistic expectations or fears.

In the following, the different forms of urinary tract reconstruction performed during childhood and adolescence such as bladder augmentation, bladder substitution, urinary diversion, or ureter replacement are described and discussed concerning their advantages and disadvantages.

## Bladder Augmentation and Substitution

A low-compliance, small-capacity bladder is the classical indication for bladder augmentation. The patient and/ or caregivers should be able to empty the augmented bladder using clean intermittent (self) catheterization (CI(S)C). If catheterization via urethra is difficult or impossible due to anatomical or orthopedic problems (the patient cannot easily reach the urethra to perform the CISC), a continent catheterizable stoma (“Mitrofanoff” stoma) should be offered (20). In patients with an incompetent urinary sphincter, who need a bladder neck procedure, the placement of a continent catheterizable stoma should be also discussed. In patients who need a bladder neck closure, a stoma needs to be placed either at the umbilicus or in the right or left abdomen, depending on the anatomy (position of the belly button, length of the mesentery of the appendix, position of the bladder in relation to the umbilicus etc.).

For bladder augmentation, gastric, ileal, ileocecal, and colonic segments as well as the ureter can be used (2). During the operation, it is essential that the bladder is opened widely (“clam technique”) to prevent the so-called “hourglass” phenomenon (21, 22). Unfortunately, in some cases the mesenteric arteries of the ileal segment are not long enough to reach the trigone due to the wide opening. In these cases, using a colonic segment is a better choice/ option.

As early as 1899, an ileal segment was used for bladder augmentation in patients with bladder exstrophy (23, 24). In the late 1970, ileum was increasingly used to increase bladder capacity (25). The ileal segment is detubularized and reconfigured in a U- or S-shape to form a large spherical reservoir based on the residual bladder (21, 22).

The ileocecal segment was first used in the middle of the last century for bladder augmentation (26). There is no advantage for using the ileocecal segment compared to an ileal segment. However, if the ileocecal segment is used for augmentation, the appendix can be embedded in the taenia libera and used as a continent catheterizable stoma similar to the ileocecal pouch (MAINZ pouch) (27). If ureter reimplantation is necessary e.g., due to obstruction or symptomatic reflux, the ureter(s) can be reimplanted in the terminal ileum and the ileocecal valve serves as reflux protection (28).

The sigmoid colon was already used for reconstruction at the beginning of the last century. Detubularization started in the middle of the last century (29, 30). The sigmoid colon is closely located to the bladder and in cases, in which the ileum cannot be used (e.g., due to a short mesentery, Chron's disease etc.), it can easily serve for augmentation to increase bladder capacity. Disadvantages of the use of sigmoid segments are the lower capacity, higher pressures, and lower continence rate—at least in most of the studies in patients with a neobladder (31–34). To avoid metabolic complications due to the use of intestinal segments, autoaugmentation with partial detrusorectomy or detrusormyotomy creating a diverticulum have been performed. However, the results are conflicting in the literature (35–38), and mostly those with a preoperative bladder capacity of 75–80% of the expected volume have a benefit from the operation (39, 40). Also, the seromuscular cystoplasty (41, 42)—performed also to avoid metabolic consequences/complications—has not proven to be as successful as the standard augmentation with intestine (43).

Particularly in patients with neurogenic bladder dysfunction, the choice of the intestinal segment gains importance. In patients with preoperative soft stool or occasional diarrhea, the stool frequency can increase and a new fecal incontinence may occur. The reconstruction of the ileocecal valve as part of the creation of an ileocecal pouch (MAINZ pouch) has not proved to be successful in the long term (44, 45).

Since 1978 stomach has been used for augmentation particularly in patients with short bowel syndrome and/or impaired renal function (46, 47). Common complications are hyponatremic hypochloremic alkalosis and “haematuria-dysuria” syndrome in more than 1/3 of the patients (48, 49). Furthermore, it could be demonstrated that quite aggressive secondary tumors can occur starting 10 years postoperatively (50–53). Today, gastric segments should not be used anymore—if possible—due to these serious complications.

In contrast to the use of any bowl segment using the ureter to enlarge the bladder has no metabolic consequences. This method was first mentioned in 1973 (54). Thus, theoretically it would be the best material for bladder augmentation. Unfortunately, the combination of a functionless kidney with a significant dilated ureter that is well-supplied with blood vessels is very rare. Furthermore, the re-augmentation rate in larger series could be up to 73% (55, 56).

Urinary continence cannot always be achieved by bladder augmentation alone, especially in patients with neurogenic bladder dysfunction. Thus, 14 out of 21 patients in the cohort of Kaufmann et al. (57) and 20 out of 59 patients in the study of Heschorn et al. (58) remained incontinent. Autologous slings or artificial sphincters can be used to improve continence. Implantation can may be performed simultaneous to the augmentation or delayed (42, 59).

As vesicoureteral reflux is mostly secondary, the treatment is primary related to bladder function (60). Patients with a high-grade reflux before augmentation have a higher risk for persistent symptomatic reflux after the enterocystoplasty (61) and simultaneous ureteral re-implantation in high grade symptomatic reflux, especially in those with low-pressure high-grade reflux, should be discussed.

Today, bladder augmentation is usually performed by using an ileal or sigmoid segment, depending on the surgeon's preference and experience. If the ileal segment cannot be used due to anatomical or functional reasons, the sigmoid colon can be used and vice versa. Gastric segments should be avoided due to the high complication rate. If a continent catheterizable stoma is necessary, the appendix is the method of choice.

## Continent Anal Reservoirs

Continent anal reservoirs have been the first kind of continent urinary diversion. The history of continent anal diversion started in July 1851 in London. Sir John Simon performed a fistula between the ureters and the rectum in a boy with bladder exstrophy. Unfortunately, the boy died 1 year later with multiple ureteral stones and obstruction of the upper urinary tract. In October 1851, Mr. Lloyd—as well from London—performed a similar operation in a boy with exstrophy, who died 8 days later due to peritonitis (62, 63). These two first cases demonstrate the problems of urinary diversions performed in these days—infection and obstruction. Due to these problems, different kind of anal reservoirs have been created, such as the Maydl procedure, the Gersuney, the Heitz-Boyer and Hovelacque or Mauclaire bladder as well as their modifications (64–72). At the beginning of the last century, anal reservoirs had been the only option for a continent urinary diversion. In the 1930s and 40s, the ureterosigmoidostomy was used for continent urinary diversion, especially in patients with malignant disease (73). Due to the high number of surgical and non-surgical complications and consequences as well as the increased risk of secondary malignancies, this type of urinary diversion fell into disrepute (73, 74). At about the same time, Eugene Bricker popularized the ileal conduit as an incontinent form of urinary diversion—the so-called “Bricker Bladder” (75). To overcome the disadvantages of the classical ureterosigmoidostomy and reduces the number of postoperative febrile urinary tract infections as well to improve the continence rates Fisch and Hohenfellner introduced the rectum-sigma pouch (Mainz Pouch II), which transformed the high-pressure segment of the rectosigmoid into a low-pressure reservoir by detubularization and reconfiguration (76). As this diversion is used mostly in children and adolescents after failure of previous operations, the ureters are usually dilated. They can be safely re-implanted using a seromuscular extramural tunnel according to the procedure of Abol-Enein (77, 78).

In patients with an irreparable urethral sphincter defect and a small bladder capacity or even almost no bladder volume at all (e.g., after failure of primary bladder closure in patients with bladder exstrophy or incontinent epispadias) or in those in which the bladder must be removed (e.g., due to malignancies) a continent anal diversion using the seromuscular extramural tunnel technique for ureteral re-implantation can be offered. Basic prerequisite is a normal renal function, a competent anal sphincter and no previous or planned radiation of the small pelvis.

## Continent Cutaneous Urinary Reservoirs

After a functional or anatomical bladder loss, in patients with incompetent anal sphincter or if an anal urinary diversion is not desired, a continent cutaneous urinary diversion is an option. Beside a normal or almost normal renal function, the will to self-catheterization is an absolute precondition. The patient and/or the parents must be able to perform the CISC/ CIC. Furthermore, a continent cutaneous urinary reservoir can be applied in preparation for a kidney transplantation (79).

After the first reports about the use of the cecum with the appendix as a stoma for continent cutaneous urinary diversion in the beginning of the last century (80, 81), the idea of a continent cutaneous urinary diversion was re-discovered in the 1950s by Gilchrist and his co-workers (82, 83). Nils Kock introduced the principle of detubularization and reconfiguration of intestinal segments for continent cutaneous urinary diversion (84). This method led to the development of various forms of continent cutaneous urinary diversion (85–89). The MAINZ Pouch as mixed Augmentation of Ileum and Coecum uses either the submucosally embedded appendix vermiformis (27, 90) or an ileal invagination nipple with fixation in the ileocecal valve as the continence mechanism (91). The continent stoma is attached to the umbilical funnel or to the lower right abdominal wall and offers good cosmetic and functional results.

Specific complications in continent cutaneous urinary reservoirs involve the continence mechanism, the pouch and the ureteral reimplantation. For the continence mechanism three different principles have been used so far. First of all, the “flap-valve” principle and its modifications are the most commonly used techniques. Better known as the flap-valve technique under the term “Mitrofanoff” stoma, which was first described by Verhoogen in 1908 and popularized by Paul Mitrofanoff in 1980 (20, 80). The technique of Yang-Monti, is used, if the appendix is already removed or too short or obliterated (92–94). In the long-term (7.7 years), it has been shown that the complication rate of the Yang-Monti technique is significantly higher compared to the use of the appendix (95). Other authors failed to confirm the higher complication rate with a slightly shorter follow-up (5.8 years) (96). Another option is the plication of the terminal ileal segment as it is used in the Indiana pouch (87). Ardelt and coworkers demonstrated in their review, that, on average, 87% of patients are continent when using the flap-valve principle. Problems with the catheterization occurred in about 20%. Stomal stenoses are a major problem in more than half of the patients (97). The relatively high rate of easy-to-treat complications seems to be the price for a good continent stoma (98).

Secondary, the principle of “Nipple Valve” goes back to studies of Watsuji and Perl (99, 100). Kock was the first to use the “Nipple Valve”-principle in the Ileum Pouch (Kock Pouch) (84, 101). It turned out, however, that the construction is quite complicated. After a median follow-up of “only” 6.5 years, Abd-el-Gawad et al. reported pouch-related complications in 10 out of 13 children and 3 out of 7 adolescents (102, 103). If the principle is transferred to the ileocecal pouch and the invaginated nipple which is additionally fixed in the ileocecal valve, the complication rate is reduced (91). Wiesner et al. showed that ~8–10% of the patients need a revision due to stomal incontinence and 15–20% developed a stomal stenosis in the long run. This was significantly less compared to the use of the appendix (104). This may be due to the larger diameter of the stoma. In their meta-analysis, Ardelt et al. showed that continence rates are comparable to those of flap-valve mechanisms (~87%), fewer catheterization problems, and significantly lower rates of revision (97). Thirdly, hydraulic valves have not been proven to be useful in the long run (97, 105, 106).

Beside stomal stenosis, stone formation in the reservoir is one of the most common complications in children and young adults. For example, 15% of children and adolescents who have an ileocecal pouch (MAINZ pouch) due to a neurogenic bladder developed stones within the pouch after a moderate follow-up of 8.7 years (107). After performing a Kock pouch, the incidence rises to more than 40% (108). Regular and generous irrigation of the pouch can probably reduce the rate of stones (109). The third most common pouch-related complication is the development of a stenosis at the ureteral re-implantation site. Somani and coworkers demonstrated in their meta-analysis, that there is an incidence of implantation stenosis in these patients between 5 and 11% (110). Severely dilated ureters have even a higher risk of obstruction. In these patients the ureteral implantation technique of Abol Enein seems to be of advantage (111, 112). After a mean follow-up of 8.7 years, in 65 children and adolescents with 118 renal unit 16% of the submucosally implanted ureters had a ureteral stenosis and only 3% of the ureters implanted after the technique of Aboul Enein. At the last follow-up, 96% of the renal units showed in the ultrasound a reduced or a stable dilatation of the upper urinary tract (107).

If bladder augmentation with or without bladder outlet procedure is no option, the creation of a continent cutaneous urinary diversion is definitely an option in those patients who are able and willing to perform CISC. The relatively high complication rates of these complex procedures concerning the stoma, the reservoir, and the ureteral implantation site needs to be considered. These procedures should be only performed in centers of expertise for urinary diversions.

## Incontinent Urinary Diversion

Incontinent urinary diversion should be considered in patients who are not willing or unable to perform a CIC as well as patients with upper tract deterioration. Furthermore, those with an impaired renal function, who are not ready or suitable for a renal transplantation. Especially in those patients with a low or almost no compliance to CIC and/or medical therapy, a conduit is a temporary or a permanent solution. In children and adolescents, the colonic conduit has been shown to have less complications compared to the ileal conduit (113–124).

## Follow-up

In addition to the urinary diversion-related complications mentioned above, the use of bowel segments for urinary diversion may also result in metabolic changes. This is due to the incorporation of intestinal segments into the urinary tract (19). Therefore, lifelong regular follow-up is required. In this case, the upper urinary tract must be monitored by means of ultrasound and, if necessary, MAG-III clearance (assessment of bilateral renal function and exclusion of any urodynamically relevant urinary tract dilatation). Stones in the reservoir can be detected by ultrasound. Regular follow-up visits should be used to detect and treat urinary obstruction or small pouch stones at an early stage. When intestinal segments are incorporated into the urinary tract reconstruction, this absorption surface is lost to the physiological function of the gastrointestinal tract. The intestinal tract contains intrinsic absorptive and secretive properties that remain even after incorporation into the urinary tract (19, 125). A decreased absorption of vitamin B_12_ from the small intestine or a decreased reabsorption of bile acids in the small intestine as well as in the large intestine can result (19). A variety of factors determine the extent of metabolic changes: length and type of intestinal segments used for reconstruction, atrophy of the intestinal mucosa as a result of chronic urinary diversion, renal and hepatic function, patient's age, previous radiotherapy or chemotherapy, and co-morbidities of the patient (125). Changes in the acid-base or electrolyte balance occur more often after continent urinary diversion due to the longer time the urine remains in the reservoir as well as the significantly larger absorptive surface. The variations depend on the type of bowel segment used (19). The risk of developing secondary malignancies seems to be lower in continent cutaneous and orthotopic urinary diversion than in anal urinary diversion (126). Higuchi et al. showed that the incidence of bladder cancer was not significantly increased in patients after ileum or colon bladder augmentation compared to a control group (4.6 vs. 2.6%). However, immunosuppression, transplantation and smoking do appear to confer an increased risk of malignancy in the setting of the augmented bladder (127). Even at low incidence, lifelong follow-up is essential. Especially after an anal urinary diversion, a regular endoscopic examination should be performed starting the 10th postoperative year.

## Conclusion

Nowadays, bowel segments can be used safely for urinary tract reconstruction. The operative decision should be in alignment with the patient's clinical condition as well as the individual's informed choice after all options have been thoroughly presented. These complex operations should be performed in high volume institutions/ centers of expertise who could deal with the possible complication and guarantee a life-long follow-up.

## Author Contributions

RS, KZ, and NH writing and editing the manuscript.

### Conflict of Interest Statement

The authors declare that the research was conducted in the absence of any commercial or financial relationships that could be construed as a potential conflict of interest.
